# The lateral cervical stria approach to selective neck dissection: a preliminary study

**DOI:** 10.4317/medoral.25802

**Published:** 2023-01-15

**Authors:** Yiyan Qian, Zhongle Tian, Bing Li, Yanbin Xu, Yuli Wang, Yifei Du, Yifeng Bian

**Affiliations:** 1 Jiangsu Key Laboratory of Oral Diseases, Nanjing Medical University, Nanjing, People’s Republic of China; 2 Nantong Tongzhou People's Hospital, Nantong, People’s Republic of China; 3 Yancheng Third People's Hospital, Yancheng, People’s Republic of China; 4 Department of Oral and Maxillofacial Surgery, Affiliated Hospital of Stomatology, Nanjing Medical University, Nanjing, People’s Republic of China

## Abstract

**Background:**

This study aims to propose a lateral cervical stria approach for selective neck dissection (SND) in patients of early-stage oral malignancies.

**Material and Methods:**

The lateral cervical stria approach was used in 11 patients undergoing SND between December 2020 and March 2022. The surgical incision was located in submandibular cervical stria, with a length of 5.0 cm. The ipsilateral SND was performed according to the pathological type, covering part or all of I-V levels. Perioperative variables including operation time, blood loss, drainage volume, number of lymph node as well as complications were assessed. The score of appearance using the University of Washington Quality of Life Questionnaire (UW-QOL) was recorded 6-month postoperatively.

**Results:**

Direct closure of primary lesion was performed in ten patients and a forearm free flap reconstruction was used in one patient. No wound breakdown or infection was found in all cases. The mean operative time of SND was 157.63±27.39 min. The volume of intraoperative blood loss and postoperative drainage was 120.45±36.77 ml and 314.09±98.82 ml, respectively. The mean number of retrieved lymph nodes was 17.89±6.03 (ranging from 12 to 31). Postoperative complications included mild static lower lip deviation (n=1), shoulder discomfort (n=1) and mild auricular paraesthesia (n=1). The mean score of appearance was 86.36±13.06, with 100 scores in 5 patients and 75 scores in 6 patients.

**Conclusions:**

The lateral cervical stria approach for SND in early-stage oral malignancies is reliable, achieving to satisfactory functional and aesthetic outcomes.

** Key words:**Lateral cervical stria, selective neck dissection, oral malignancy, aesthetic evaluation.

## Introduction

Neck dissection (ND) for early-stage oral squamous cell carcinoma (OSCC) could screen out cases of occult cervical lymph node metastasis and improve the overall and disease-free survival (1). For patients with clinically node-negative oral cancer, prophylactic selective neck dissection (SND) is recommended for its oncological effect similar to radical neck dissection, but the sternocleidomastoid (SCM) muscle, the internal jugular vein and the spinal accessory nerve are preserved to optimize functional outcomes.

The conventional approach of SND is the submandibular approach which starts from the submental region to the mandibular angle, paralleling to the lower edge of the mandible. This approach has a clear surgical vision but leaves a long and prominent scar on the neck affecting the aesthetic outcome. With the trend towards minimally invasive and aesthetics, remote approaches or small incisions to perform SND have been descried in previous studies, mostly assisted by endoscopy or surgical robots (2-4). However, due to complexity of operative techniques and anatomical architecture of the neck, endoscopic- or robotic neck dissection could not be routinely practiced without extra specific long-term training (5).

The cervical stria is a normal dermatoglyphic structure of the neck. It is reported that lymphadenectomy of all levels could be completed through the transverse cervical stria approach in the neck, achieving to favorable cosmetic and functional outcomes (6,7). Fan *et al*. investigated the lateral neck approach for endoscopic-assisted selective neck dissection in patients of early-stage head and neck squamous cell carcinoma (2). However, the lateral cervical stria approach to SND in oral malignancies without endoscopic- or robotic assistance has not been reported yet.

The purpose of this study aims to investigate the reliability and safety of the lateral cervical stria approach to SND for early-stage oral malignancies as well as its atheistic and functional outcomes. To the best of our knowledge, this is the first study using a small incision (about 5 cm in length) in lateral cervical stria to SND without assistance of endoscopy or surgical robots.

## Material and Methods

- Patients’ selection

From December 2020 to January 2022, a total of 11 patients diagnosed as early-stage oral malignancies at the Department of Oral and Maxillofacial Surgery, Stomatological Hospital affiliated to Nanjing Medical University was retrospectively reviewed. All cases were clinically and pathological examined to confirm the primary lesions. Inclusion criteria were as follows: pathological diagnosis of oral malignant tumors requiring ipsilateral SND; no evidence of cervical lymph node metastasis based on clinical and radiographic examinations; no surgical history or traumatic scar in the neck; body mass index (BMI) was less than 25.0.

- Technique

**Figure 1:**
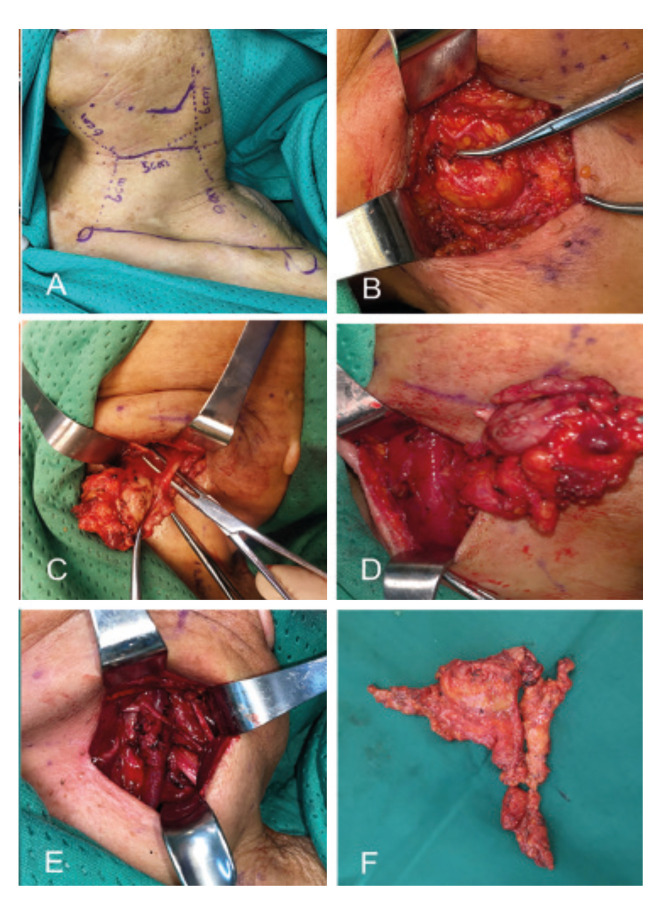
The procedure of lateral cervical stria approach to SND. A: The location of the incision of lateral cervical stria approach; B: The explosion of marginal mandibular branch of facial nerve; C: The dissection of submandibular gland and ligation of proximal end of facial artery; D: Pulling the levels I-III dissected tissues out of the incision to obtain adequate room for clearing levels IV and V; E The spinal accessory nerve, internal jugular vein and cervical plexus; F The dissected tissue of SND. Fig. 2: Appearance of the lateral cervical stria approach intraoperative (A), 3- and, and 6-month postoperative (B-D).

The primary lesion was resected under general anesthesia with negative pathological margins. The ipsilateral SND was performed according to the pathological type, and the scope of dissection was part or all of I-V levels. The head deviated to contralateral side, and the lateral cervical stria of the upper neck was selected as the surgical incision. The incision was below the submandibular region with a length of about 5.0 cm, locating in the middle of lymphadenectomy area (Fig. 1). The flap was raised in a subplatysmal and supra-venous plane, and was further elevated with deep retractors, going forward to the submental region, backward to the posterior edge of the sternocleidomastoid muscle, upper to the lower edge of the mandible, and lower to the intersection of the omohyoid muscle and the internal jugular vein. During the clearance of level I, the mandibular marginal branch of the facial nerve as well as the greater auricular nerve were identified and preserved (Fig. 1).

The proximal end of facial artery was dissected deep to the posterior belly of the digastric and ligated at the inner part of the gland (Fig. 1). After completing level I dissection, the dissected tissues were pulled out of the incision to create adequate working space for levels II and III, as well as levels IV and V if necessary (Fig. 1). Effective deep traction was significant to address level II and III regions, and the electrotome and harmonic scalpe of long tips facilitated the clearance of fibrofatty tissue in these levels. Absorbable sutures and double click electrocoagulation were used for hemostasis. The internal jugular vein, spinal accessory nerve and cervical plexus were preserved and the entire neck dissection was completed (Fig. 1).

The operation time, bleeding loss, number of retrieved lymph nodes, postoperative drainage volume and other relevant data were collected. Postoperative complications such as nerve damage and shoulder dysfunction were reviewed 6-month after operation. The appearance of neck was assessed using the University of Washington Quality of Life Questionnaire (UW-QOL).

## Results

The demographic and clinical data of the patients were given in Table 1. A total of 11 patients was enrolled in this study consisting of 9 cases of OSCC, one case of adenoid cystic carcinoma (ACC) and one case of mucoepidermoid carcinoma (MEC). There were five females and six males in the series, with a mean age of 58.09 years (ranging from 29 to 86 years). The mean BMI was 23.27±1.42. The primary sites included tongue (*n*=5), buccal (*n*=3), submandibular gland (*n*=2) and upper gingiva (*n*=1). Among 9 OSCC cases, 4 and 5 cases had T1 and T2 primaries, respectively. The cases of submandibular gland ACC and MEC received supra-hyoid (levels I–III) and supra-omohyoid (levels I–IV) neck dissections, while 9 OSCC cases received expanded supra-omohyoid neck dissection (levels I–V). Pathologic N1 status was confirmed in two cases (the submandibular gland MEC case and one OSCC case) who were recommended to undergo postoperative radiotherapy; however, the patient of submandibular gland MEC refused.

**Table 1:**
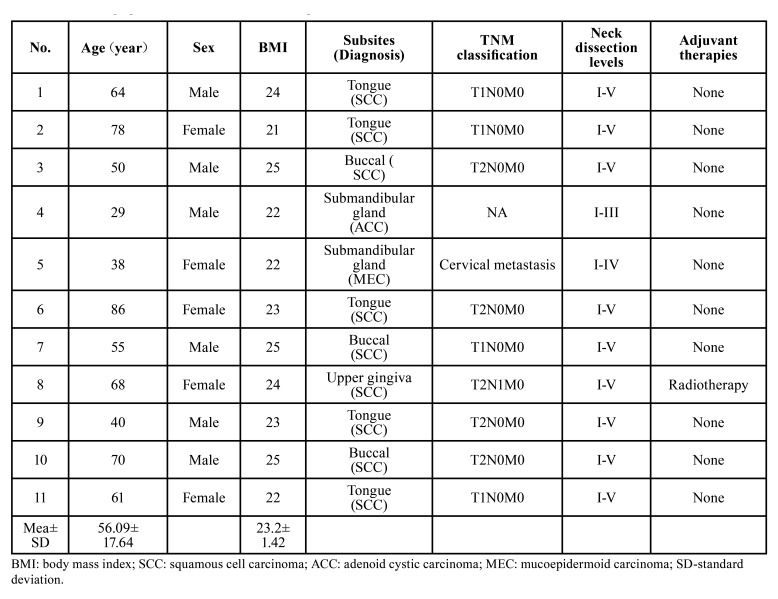
The demographic and clinical data of enrolled patients.

**Figure 2:**
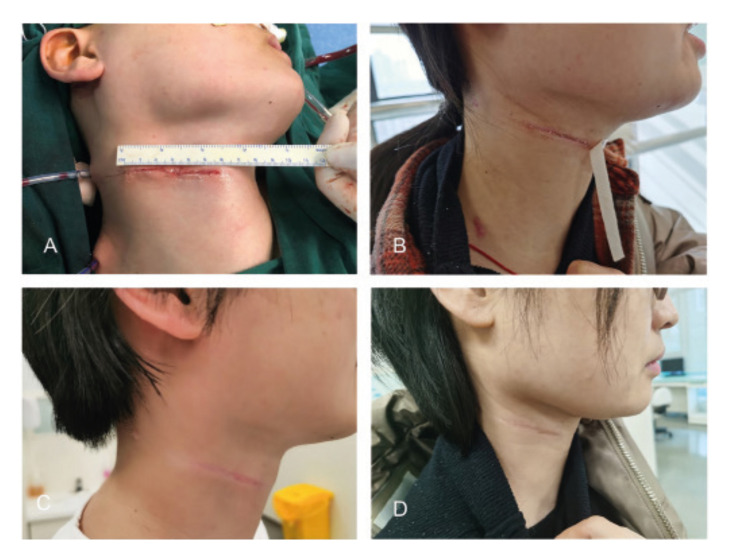
Appearance of the lateral cervical stria approach intraoperative (A), 3- and, and 6-month postoperative (B-D).

Direct closure of primary lesion was performed in ten patients and a forearm free flap reconstruction was used in one patient. No wound breakdown or infection occured in all cases. As shown in Table 2, the mean operative time of neck dissection was 157.63±27.39 min. The volume of intraoperative blood loss and postoperative drainage was 120.45±36.77 ml and 314.09±98.82 ml, respectively. The mean number of lymph nodes was 17.89±6.03 (ranging from 12 to 31). The average follow-up time was 15.5±6.28 months. Postoperative complication included mild static lower lip deviation (*n*=1), shoulder discomfort but no limitation of arm lifting (*n*=1) and mild auricular paraesthesia (*n*=1). The mean score of appearance was 86.36±13.06, with 100 scores in 5 patients and 75 scores in 6 patients. The representative case was shown in Fig. 2, achieving a satisfactory appearance of the lateral cervical scar 3- and 6-month postoperatively.

**Figure 3:**
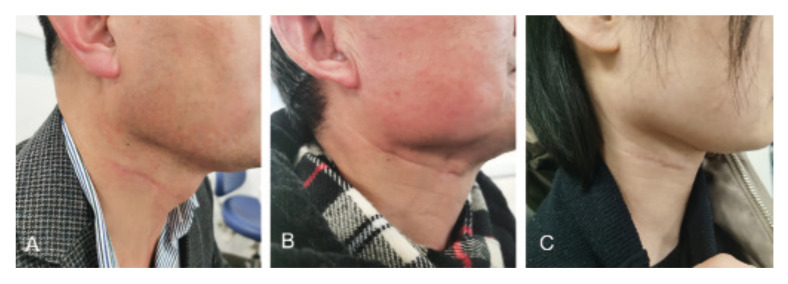
Three types of the lateral cervical stria showing 1(A), 2(B) and 3 (C) lines of wrinkles.

The number and location of the upper cervical striae of the lateral neck vary from person to person. Among included 11 cases, three types of cervical stria on the upper lateral neck including 1, 2 and 3 lines of wrinkles (Fig. 3). Except for cases of 1 line of wrinkle, the second line of lateral cervical stria was routinely chosen for SND.

**Table 2:**
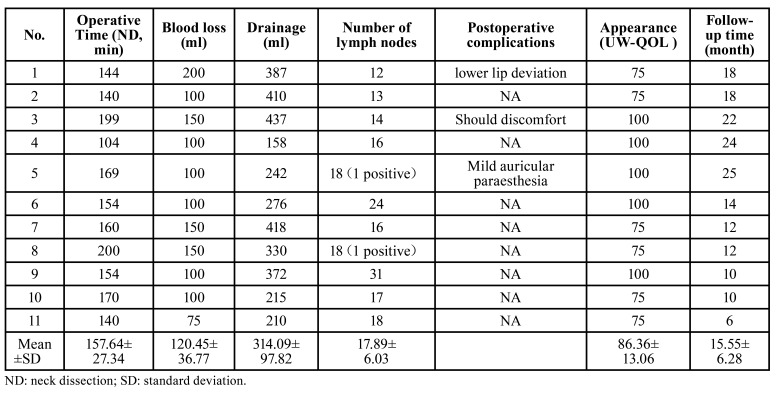
The perioperative and follow-up data of enrolled patients.

## Discussion

The surgical approaches to neck dissection may vary, due to institution’s tradition, personal preference as well as dissected levels. Besides functional and oncological considerations, more and more surgeons place an emphasis on reducing aesthetic impairment of the neck. To avoid extensive visible neck scars and improve cosmesis, two maneuvers are addressed in the previous literature: (1) remote approaches, for example robotic assisted retroauricular approach (4,5); (2) transverse cervical approach hidden in the natural creases of the neck (6,7), or small lateral incisions, usually assisted with endoscopy (2,3). The retroauricular approach is considered as a routine approach of endoscopic- or robotic-assisted SND for oral cancer as well as thyroid carcinoma with lateral neck metastasis. Documented studies demonstrate the superior aesthetics of the retroauricular approach compared with conventional submandibular approach; however, the cost of prohibitive equipment and long training cycle refrain the clinical application of endoscopic- or robotic-assisted neck dissection (5,8,9). Moreover, inadequate access to level Ia via the retroauricular approach challenges the oncological safety of submental clearance. Recently, Kudpaje *et al*. presents a novel transoral retroauricular neck dissection (TREND) that overcame these factors but need an additional buccal vestibular incision which makes the intraoral and neck incision communicating (10). In patients of early-stage oral cancers, the primary lesion is commonly addressed transorally, necessitating a separate incision on the neck for SND.

Transverse cervical approach hidden in the natural creases of the neck is described by Langer and is widely accepted with the purpose of reducing aesthetic impairment (6,7,11). The lateral submandibular approach to endoscopic SND (incision length ranging from 3.0-5.5 cm) is reported in patients of early-stage head and neck SCC (OSCC included) and lateral cervical metastases of thyroid papillary carcinoma (2,3,12). Inspired by these studies, we attempt to select the lateral cervical stria on the upper part of neck for SND in early-stage oral malignancies with routine surgical instruments including long cautery tips and deep retractors, meeting the aesthetic expectations without compromising the oncological outcome. The psychological stigma induced by the residual unaesthetic scar after neck dissection affects the patient’s quality of life (13). In this series, the mean score of appearance is 86.36±13.06, with 100 scores in 5 patients and 75 scores in 6 patients, achieving a satisfactory appearance of the lateral cervical scar postoperatively.

For most oral cancers, the traditional incision design needs to extend close to or even beyond the middle of the neck, so as to better expose level I and II. The incision described by Langer is approximately 7.0 cm, starting from the upper outer edge of the thyroid cartilage to the posterior border of the sternocleidomastoid muscle (6,11). The submandibular small incision used to complete endoscopic SND for early oral cancer (level I-III) and thyroid cancer (level II-VI) is 3.0~5.5cm in length (2,3). The length of lateral cervical stria incision in the present study is about 5.0 cm, locating in the carotid triangle area, which was closer to level Ia in contrast to the endoscopic SND incisions. Levels Ib, II and III ae easy to access while levels IV and V are exposed by retracting of deep hooks. While working in such a confined space, two critical factors should be mentioned. Firstly, the separation of the platysma should be as widely as possible so as to increase the mobility of the skin flap. Secondly, pulling the levels I-III dissected tissues out of the incision to obtain adequate room for clearing levels IV and V, is a key step during neck dissection. Hence, levels I-V areas can be reached under direct vision without need of endoscopy. However, due to limited working space, surgeons need to be familiar with the cervical anatomic architecture and well experienced in neck dissection. Even so, the mean operative time of neck dissection is more than 2 hours, which is longer than that of previous studies (2,3,6). Additionally, be caution to choose obese patients as indication of the lateral cervical stria approach. In the present study, BMI of each patient was less than 25.0.

Some researchers believe that cervical nodal yield is a robust independent prognostic factor in cN0 OSCC patients undergoing elective neck dissection (END) and suggested 18 is a minimum accepTable nodal yield (14). However, the mean number of lymph node differs in previous studies. It is reported that using transverse extended incision in neck dissection could isolate an average of 18 ± 7 (ranging from 11 to 31) versus 16 ± 5 (ranging from 6 to 29) of control group (7). Another report presents averaged 21.7 (ranging from 10 to 51) nodes per dissection in the ‘‘Paul André’’ group against 22.6 (ranging from 11 to 49) nodes per dissection in the “transverse cervicotomy” group (6). The mean number of lymph node in the current study is 17.88 ± 6.45, but retrieved lymph nodes in 6 of 11 patients was less than 18, indicating inadequate lymphadenectomy may be performed. However, all these patients were followed up more than 10 months, and no occurrence of postoperative cervical lymph node metastasis was found.

The second cervical striation in the central region is usually selected for neck dissection, but the location and number of the lateral cervical stria are irregular. Within the limited included cases, we observed three types of cervical stria on the upper lateral neck, varying from 1 to 3 lines of wrinkles (Fig. 3). Except for cases of 1 line of wrinkle, the second lateral cervical stria is routinely chosen due to the following considerations: 1) Avoid being too close to the lower mandibular edge, which may easily damage the marginal mandibular branch of the facial nerve; 2) With respect to the levels IV and V dissection, the second lateral cervical stria is more likely to expose the spinal accessory nerve as well as lower parts of the internal jugular vein. These important architectures locate in the deep of lateral neck rich in blood vessels and nerves, and are easy to be damaged if not handled properly. In this study, all cases presented accepTable intraoperative blood loss and postoperative drainage volume without nerve injury, indicating that SND via lateral cervical stria approach is safe and reliable.

As a retrospective analysis, this study did have many shortcomings. All the cases included in this article are early oral malignant tumors. For the cases with evidence of metastatic lymph nodes, the lateral cervical stria approach is not recommended. However, even though clinical and imaging examinations do not support cervical lymph node metastasis, there is still a proportion of nodal positive cases. These patients should be closely followed up or added with further radiotherapy. The number of cases in this study is limited and the follow-up time of three cases is less than 12 months. However, in terms of short-term observation, the lateral cervical stria approach to SND is safe and feasible, and the neck scar hidden in the natural crease is concealed and cosmetic.

## Conclusions

Within the limitations of the study, the lateral cervical stria approach to SND in patients with early-stage oral malignant tumors is reliable and has good aesthetic outcomes, which could be superior to conventional submandibular approach in appropriate cases.
